# Coming to Terms with the Legacies of the Pound Model in Animal Sheltering in the United States

**DOI:** 10.3390/ani14091254

**Published:** 2024-04-23

**Authors:** Katja M. Guenther, Kristen Hassen

**Affiliations:** 1 Department of Gender and Sexuality Studies, University of California, Riverside, CA 92521, USA; 2 Cummings School of Veterinary Medicine, Tufts University, Medford, MA 02155, USA

**Keywords:** animal sheltering, companion animal welfare, dogs, cats, path dependence, animal shelter history

## Abstract

**Simple Summary:**

In spite of significant reductions in the numbers of animals entering shelters and being killed there over the last decades, the beliefs that companion animals must be under the direct control of a human guardian, and that capturing and often killing animals is morally justifiable and even necessary, continue to guide animal sheltering practices in the United States. Looking at the historical origins of animal control and sheltering in the US reveals a high level of consistency in ideologies and practices across almost 150 years. In this commentary, two scholars on animal sheltering in the US examine the historical legacies of animal control and sheltering in the US to show how they are problematic and dysfunctional and need to be abandoned to improve the lives of companion animals and the human communities of which they are a part.

**Abstract:**

This paper examines the legacies of the emergence of the animal control and sheltering industry in the United States and their impact on contemporary public animal shelters. While decades of gradual reform have helped substantially reduce the number of animals entering shelters and being killed there, contemporary animal sheltering largely continues to follow the path set when animal sheltering developed in the late 1800s and early 1900s. Three key interrelated legacies of the pound model of early animal control and sheltering enduringly shape sheltering today: (1) the institutional culture of animal shelters grounded in the logics of caging and killing; (2) the lack of visibility and transparency, especially within government shelters; and (3) the economic logics of the pound model, including the disparities in sheltering resources across communities. Examining the origins of animal control and sheltering and identifying the specific legacies of this pound model within contemporary government-funded shelters improves understanding of why such shelters in the US have developed with a particular set of practices and ideologies, and thus provides an important footing for envisioning and enacting radical changes in animal sheltering.

## 1. Introduction

The historical continuities in the practices and ideologies of public animal sheltering in the United States need to be addressed in order for sheltering to move permanently away from killing as a so-called solution to the problem of free-roaming and homeless companion animals. The model of animal control and sheltering that emerged in the late nineteenth century in the US continues to define how nearly all the largest public animal shelters in the US operate today. In this commentary, we present a reading of the development of animal sheltering in the US that challenges the widespread belief that shelters came into being primarily to combat the spread of rabies. We instead show how the actual bases for the pound model continue to inform the principles and practices of animal shelters today. The pound model of managing populations of unhoused or otherwise surplus cats and dogs centers on a pair of ideas, namely that companion animals must be under the direct control of a human guardian and that capturing and often killing animals is morally justifiable and even necessary. While animal shelters have made important changes, and have adopted new monikers, such as “care center” or “shelter”, many of the underlying principles of the pound model remain largely unchanged.

The term “pound model”, which we detail below, describes the dominant model of government-funded animal services in the United States. Though the term has a pejorative association for some participants in the companion animal welfare industry (along with terms like “dog catcher”), invoking the pound model reflects the historical roots of the modern impound facility, modeled after the English penfolds in which stray livestock were confined and owners could reclaim their animals for a fee or their animals would be sold at auction [1]. This model was adapted for dogs and cats who, unlike farmed animals, did not have market value and were typically culled if unclaimed at the end of the hold period.

Social and economic scientists studying institutional change developed the concept of path dependency in part to refer to how setting down one path shapes and constrains future decision-making [2]. That is, the path we are on influences which paths we will continue on, and how we do so. As a simple example, if we start down a path heading due East, and become accustomed to following the pattern of the sun in a particular way because we are always moving East day after day after day, changing direction to travel North, South, or West may feel confusing, overwhelming, or even like backtracking. We thus are likely to continue to move East. Further, while moving East, we may develop specific logics and biases to justify our movement direction (“we move towards the rising sun”) and practices (such as celebrating the rising of the sun each day or moving primarily in the earlier part of the day, when the eastward pull of the sun is clearest). These practices, and the ideologies that emerge to support them, ultimately make it increasingly difficult for us to justify or implement a change in direction, even when there may be a clear benefit to doing so.

Path dependence helps us make sense of why, even when there is sometimes a slight shift in practices and policies, the path pulls institutions back onto it. Path dependence is thus a useful framework for thinking about the consistency of the pound model over time. As we detail in this commentary, the pound model is the path that animal sheltering in the US has followed since the late 19th century, and while there have been efforts to move the path somewhat, external stressors contribute to shelters in the US remaining on the same path. When placed under the strain of worsening economic conditions, crises in local governments, and disruption caused by a temporary suspension and restarting of the system of animal control impoundment and disposition of animals due to the COVID-19 pandemic, government shelters readily revert back to killing as a “solution” [3]. In other words, the institution of animal sheltering in the US, which finds itself in crisis, is following the same path set at the advent of animal control. In 2023, stray dog intakes went up 30% from 2021 and the killing of impounded dogs doubled compared to 2021 [3]. Meanwhile, adoptions and transfers to rescue groups remain significantly lower than before the pandemic. The resultant gap between intakes and outcomes means shelters are overcrowded and more animals are losing their lives to make space for incoming arrivals.

This is a critical moment of opportunity to examine the path that the shelter system in the US followed to arrive at this point and to envision alternative pathways forward that involve a complete break from the pound model. We need first to understand the development of the pound model and identify its problematic legacies so that we can start to map new paths forward. We also need to recognize that other ways of doing things are possible. In the field of companion animal welfare, trap/neuter/return (TNR) is one powerful example of disrupting the pound model. Through TNR, community (formerly known as feral) cats are trapped, sterilized, and returned to their colony. TNR provides services to cats without impoundment, generally free from the logics of both caging and killing.

Throughout this paper, we analyze problems that are built into the US system of animal sheltering, which emerged during the mid-19th century. Our critique is of the system itself; it is not our intention to provide commentary on specific organizations, shelter staff or volunteers, or local governments. Countless dedicated and kind-hearted people have worked throughout the last 175 years to reform and improve animal shelters. Many of those who helped to reform animal shelters over the past 30 years remember when mass killing was the norm and are terrified of returning to that past. Their fears are justified: If we do not urgently address the root causes that led to killing as the norm in animal sheltering, we are at risk of repeating this history. For that reason, we are writing this commentary as a call to action at a time when rates of shelter killing have markedly increased and the gains of recent decades are stalling and even reversing [3,4].

In what follows, we explore the origins of the U.S. animal shelter system and why the pound model remains so enduring, despite decades of reform efforts. We begin with a brief discussion of the diversity of animal shelters in the US that includes a review of key terminology used in this analysis, before turning to a more detailed history of the development of the pound model. We then disentangle three of the key interrelated legacies of the pound model: the institutional culture of animal shelters grounded in the logics of caging and killing; the lack of visibility and transparency within government shelters; and the economic logics of the pound model, including the disparities in sheltering resources across communities. Examining the historical origins of the pound model and identifying the specific legacies of this model within contemporary government shelters improves our understanding of why companion animal welfare in the U.S. has developed with a particular set of practices and ideologies that extend far beyond shelters themselves and into our public beliefs about animal shelters, animal control, and the animals that are both the driving force of the system and the problem that disrupts it.

Our analysis draws both on historical research and on our experiences within the animal sheltering system in the US. Guenther has spent close to a decade conducting research on companion animal welfare and has particularly in-depth knowledge of public animal shelters and community cat programs in southern California e.g., [5,6,7,8], one of the nation’s highest intake and highest kill rate per capita areas. Over more than a decade, Hassen has directed three large public shelter systems and is a national leader of novel methods to reduce shelter intakes and shelter killing. Tallied together, the authors have engaged in research and/or evaluations at over one hundred animal shelters in all regions of the US except Alaska. Here, we draw on historical evidence and our extensive knowledge from working in the US animal sheltering industry to illuminate the problematic historical legacies of the pound model.

## 2. Conceptualizing the Pound Model and Animal Shelters

We employ the concept of the pound model to refer to an approach to companion animal welfare that, dating to its inception in the mid-eighteenth century, holds that (1) free-roaming companion animals pose a threat to themselves and others and must be contained; (2) local governments or government-contracted agents must develop and enforce regulations pertaining to companion animal welfare, including vaccination requirements, leash laws, etc.; (3) local facilities with cages should be used to house unwanted companion animals and those who have been impounded because they were roaming free or they or their guardians violated regulations; and (4) facilities housing stray or unwanted companion animals must kill some proportion of impounded animals in order to manage the population of companion animals in and out of the shelter. In the contemporary United States, the pound model is usually at work in facilities commonly referred to as animal “shelters”, which may include publicly funded impoundment facilities operated by animal control departments, privately funded non-profit humane societies, and private organizations that are contracted by local governments to operate animal services.

Pounds and animal shelters are physical places in which animals are housed, whereas animal services are the agencies and organizations that encompass the range of programs provided by local government entities. Animal services agencies, some of which are non-profit organizations, often oversee animal control, licensing, TNR programs, and code enforcement responsibilities. In the US alone, there may be as many as 4000 animal shelters and animal control agencies and organizations [9]. Many of these agencies have contracts with local governments (such as municipalities or counties) to provide confiscation, impoundment, animal care, live release, and euthanasia [5]. The budgets, animal intakes, and staffing vary dramatically from across agencies and organizations. One animal shelter may take in 30 animals annually while another takes in 30,000 animals. One may have 2 staff members while another has 200. One may have a live release rate of 99% and another may have a live release rate of 25%. Operationally, shelters and animal control agencies and organizations also vary dramatically, depending on factors unique to each community such as local ordinances, laws, and regulations, the department that oversees the animal shelter, the level of local government support for animal welfare, the individual philosophical orientation of the animal shelter leadership, funding streams, and the culture of each agency or organization. Recognizing the diversity inherent in this grouping, our focus in this paper is on taxpayer-funded government shelters that provide contracted animal control and sheltering services to government agencies, most often in one or more municipalities. Open-admission shelters (although a growing number seem to have moved towards so-called managed intake during the COVID-19 pandemic) that are either wholly public institutions or are government agencies or not-for-profit organizations holding public contracts for impoundment and/or sheltering constitute roughly 81% of shelters in the US [10]. Although rescue organizations play a significant role in companion animal welfare in the US, and may be included in some data pertaining to animal sheltering, they are distinct from the facilities at the center of this commentary because they do not typically impound free-roaming animals, and, if they take surrendered animals, they have a closed admission system. Unlike most human services, animal sheltering does not yet have a consistent methodology for counting animal shelter intakes and outcomes, which complicates the ability to make general statements about the numbers of animals in US shelters or their outcomes [11]. We thus are, at best, estimating that around 6 million animals enter US shelters annually and 1.5 million are dying, being euthanized, or being killed there.

A paper addressing the pound model must necessarily discuss the killing of companion animals. Throughout this commentary, we use the term “euthanasia” according to its dictionary definition as the act of ending the life of an untreatable and irremediably medically suffering being [12]. “Kill” more accurately describes the act of the shelter taking the life of any animal who is not medically suffering with a dire prognosis [5]. Of course, standards for which animals are and are not savable and treatable are subjective and non-standardized. For example, a diabetic cat in one shelter may be considered medically suffering and untreatable, and in another shelter may be viewed as having a treatable or manageable medical condition. Agreeing upon what constitutes a healthy, adoptable animal is a source of contentious debate, as these terms are often unclear and are used to provide cover for killing shelter animals. In 2004, for example, the last time the animal sheltering industry came together to attempt to standardize intake and outcome reporting, the resulting Asilomar accords loosely defined terms like “untreatable”, and “unmanageable”, asserting that animals in these categories should not be included in a facility’s reported live release rate [13]. We further recognize the debates surrounding ending the lives of animals who are deemed behaviorally unsafe to be around humans; still, as these animals are not typically suffering per se, ending their lives is also killing, not euthanasia. 

## 3. A Brief History of the Pound Model

Exploring the roots and development of the pound model in the United States is essential for understanding the language, laws, and norms of animal shelters in the contemporary period. Contra to perspectives that animal shelters today represent a marked rupture from the pounds of the past [14], contemporary publicly contracted animal shelters retain much from their pound forebears. Animal sheltering and control in the US emerged out of two sometimes competing impulses: the desire of municipalities to regulate animal and human populations and the demands of the broader animal protection movement that took off in the latter part of the nineteenth century in the US to reduce animal suffering and uphold human morality. Municipal pounds tended to focus on public safety and on culling animals. Animal protectionists centered moral and religious claims in their rhetoric, arguing that the abuse, neglect, vivisection, and slaughtering of animals would morally corrupt those who witnessed it or lived proximal to it. Animal protectionists advocated for the humane treatment of animals, and often took over municipal pounds and/or founded animal shelters in their communities that both sought to rehome animals and to kill animals using methods they deemed more humane than those of municipal pounds [15].

Whether a fully public, fully private, or hybrid institution, animal control agencies emerged to regulate working, farmed, and companion animals, and expected guardians of animals to adopt specific practices regarding how they related to and cared for them. Working class people and people of color who used animals, such as carriage horses, cart and plow oxen, and other “beasts of burden”, for labor, as well as animals who were members of communities rather than belonging to one specific owner (e.g., free-roaming domesticated animals), were the frequent targets of municipal animal control officers. Animal protectionists both sought to reign in animal control officers by limiting their use of violence against animals and to empower the officers in ways parallel to human police [15,16].

The advent of licensing requirements and so-called “dog taxes” in the earlier 1800s set the tone for the class-based differentiation of companion animals and their guardians that continues to this day. In cities like Washington, DC, New York, and Philadelphia, the affluent white guardians of the 19th century formed associations that worked to differentiate the animals in their care—most often purebred dogs—from so-called tramps, or street dogs, with the immediate goal of avoiding muzzling requirements for their dogs during periods of rabies panic [16,17]. Many of the nation’s most prominent humane advocates, including Henry Bergh, founder of the American Society for the Prevention of Cruelty to Animals (ASPCA), were vocal in defending purebred, owned dogs from impoundment and muzzling, even as they actively participated in culling street dogs [15,18]. From the outset, then, animal control agencies distinguished between owned dogs and so-called stray dogs and targeted the latter almost exclusively for regulation, removal, and death [19,20].

Within the US, the existence of the pound itself fundamentally shifted expectations for the role of government and the place of companion animals and other domesticated animals in our lives and communities [16]. Whereas before the late nineteenth century, various types of domesticated animals, including dogs, horses, bovines, cats, and others, were considered part of interspecies communities, living and moving among and with their caregivers, the advent of animal control and pounds reflected a growing interest in separating humans and animals, and in maintaining clear property relationships between specific people or business and specific animals. These changes necessitated that community animals, especially dogs, and later cats, be removed and destroyed in the name of public welfare and safety [16,17,21,22]. In his analysis of the embrace of specific norms around dog-keeping in modern western cities, or what he refers to as dogopolises, Chris Pearson [16] situates the growth of animal control strategies for managing stray dogs within the tensions and inequalities of modernizing cities: “With bourgeois wealth and success rooted in the free movement of individuals and commodities, stray dogs joined prostitutes, manual laborers, beggars, and hawkers as unwelcome and physical obstacles to wealthy metropolitan lifestyles. Cast as mobile, diseased, and disruptive creatures, they had become dislocated from civilized human society. Campaigns to cast them from the city became early building blocks of dogopolis”. 

At the same time, animal protectionists engaged in respectability politics that asserted that “civilized” and “moral” people practice compassion toward companion and working animals [23]. When humans violated these newer expectations of how to care for animals, they were subjected to a range of punitive and carceral responses, including loss of the animal(s) (i.e., impoundment), fines, or even jail. Black Americans, newer immigrants, poor and working class people, and the animals in their care were (and remain) overrepresented among those who animal control officers policed. Animal control was in many ways an extension of the policing models that sought to control these human populations. The result of this was that the bonds between companion animals and people were precarious, and “ownership” existed only to the extent that animal control officers allowed it, since they could confiscate and destroy any unlicensed animal at will [17].

Making the deaths of companion animals appear more humane was one of the key ways that animal protectionists helped integrate owned dogs into the social fabric of American life [16]. Many humane reformers pushed against what they saw as particularly cruel methods animal control workers used to kill dogs and cats, such as bludgeoning and drowning, and worked to establish gas, which reformers believed was more humane, as the preferred method of killing. They also improved the quality of care that apparent street animals received, both on the street and in shelters. The first animal shelter in the United States—that is, a place to hold animals while also seeking their guardians or new homes—opened in Philadelphia around 1870, and offered impounded animals food, water, and some cover [21,24]. Reflective of the animal protectionist push toward more humane methods of killing, the Philadelphia Society for the Prevention of Cruelty to Animals (PSPCA) widely promoted its evolving methods of shelter killing, sharing knowledge with other facilities and the public, and detailing why different methods were appropriate for different types of animals (cats and kittens, for instance, continued to be drowned long after dogs were being killed by gassing).

As animal control agents became more efficient at rounding up stray dogs, shelters shifted from housing large groups of dogs in shared pens towards cages intended to hold smaller numbers of animals, or even just one dog or cat per cage. (When cats were impounded, they were almost always killed immediately [15]). Animal control officers could manage animals more easily as individuals than in groups. Keeping them separately was believed to slow the spread of disease and reduce the risk of injury. Housing impounded animals in groups also posed potential liabilities for animal control and the governments that oversaw them should an animal in the custody of the state agency become injured or sick.

One of the catalysts for the early development of animal control for dogs was the public panic about rabies, a disease that became a key justification for campaigns to round up and kill free-roaming dogs in America’s rapidly growing cities and towns even in the absence of obvious risk [16,17]. Rabies, and the intense misinformation about it in the early twentieth century, played an important role in framing free-roaming dogs as a deadly menace to the public, and reinforced the idea that the sanitation practices of lower-class people contributed to the spread of diseases, which could be stopped only by policing them and their animals [16]. While less common, panics about infant paralysis also resulted in mass pound killings of cats in the early twentieth century. Though often overlooked as victims of early pounds, the ASPCA in New York City killed over 350,000 cats in 1900 alone [15].

Importantly, rabies was never a widespread disease in the US, affecting just over 100 people each year in a nation of 76 million in the early 1900s [25]. While public narratives of the founding and institutionalization of animal shelters often make rabies central, rabies was but one of many concerns that facilitated the emergence of the animal pound. Animal noise, defecation, property damage, unsightliness, and a general push to “clean up” urban environments were at least as important in supporting the emergence of the pound [16].

Today, rabies control remains a mandate of animal control officers, and the receiving, monitoring, and killing of potential rabies threats is still at the center of animal welfare laws, regulations, and policies. This is true even with little evidence to support a need for rabies management: rabies treatment is widely available and only twenty-five cases of rabies in humans were reported in the US in the decade 2009–2018, of which at least seven cases involved infection that started outside of the US [26]. While possible, there is no research to suggest that this low rate is an outcome of the activities of animal shelters. Rabies is currently used to justify animal control surveillance and impoundment, mainly of dogs, who are subject to licensing requirements that center on the rabies vaccine, even though cats are far more likely than dogs to contract rabies in the US (although both are still highly unlikely to do so—wild animals make up more than 93% of rabies cases in the US [27]). However, cats are rarely surveilled by animal control around rabies and are generally not required to be licensed at all. This inconsistency is just one example of how animal control policy does not always reflect actual conditions, and how rabies mythology remains powerful in guiding policy.

In sum, by the early 20th century, the pound model was the dominant response to managing companion animal populations in the US. The pound model relied on animal control officers (or dog catchers), as well as private citizens, to report and/or impound animals, who were then kept in cages. Impounded animals disproportionately came from poor communities. Based on the socially important role of street animals around the world today [28,29], it is not unreasonable to assume that many of those impounded as strays had bonds with humans, but such bonds were not recognized in the absence of a license or payment of a dog tax. Unless wearing a tag indicating they are licensed or clearly a purebred animal, almost all impounded animals would lose their lives after a very short stay at the pound [15,16,17].

## 4. The Institutional Culture of Public Animal Shelters

The history of the pound model in the US left an important legacy in the institutional culture of contemporary animal shelters. Even with increased pressure to rehome more and kill fewer animals, and to control the reproduction of animals who pass through shelters, many of the core ideologies and practices of early dog pounds have not evolved. Perhaps most centrally, the animal sheltering industry continues to view free-roaming animals (especially dogs) as hazards to themselves and others, and embraces impoundment as the preferred response to such animals (except for community cats in many areas). Municipal ordinances vary widely, but in most cases, ordinances still mandate impoundment in every case, and, in many cases, mandate the killing of certain types of animals, such as those not reclaimed or adopted [30]. Although somewhere between 50 and 75% of the world’s nearly 1 billion dogs are free-living (with and without caregivers) [31], the idea of dogs living among us, in communities, is almost unimaginable in most of the US.

The system of classifying animals entering pound custody also remains conceptually similar to the system used in the mid-nineteenth century, and is important in determining what happens to animals in shelters. Stray companion animals, who constitute the majority of animals who enter government-funded animal shelters in the US [32], are still understood as those who are outside the confinement of their caregiver’s privately owned or leased property. They are therefore immediately viewed as out of place and problematic. Licensing (and sometimes other forms of identification, like microchips or identification tags) remains the mechanism through which caregivers indicate their ownership and, in most cases, their successful compliance with ownership requirements, including rabies vaccination and sometimes sterilization. Across the US, state laws, municipal regulations, and/or shelter policies treat licensed and unlicensed animals differently, giving the former more time at a shelter before they can be killed. State-mandated stray hold laws vary from 24 hours to 10 days or more [33], with various exceptions that allow the state to euthanize or kill an animal prior to the expiration of the stray hold [34]. In the state of California, for instance, which has consistently been among the top two largest producers of shelter animals (along with Texas) in the US [34,35], impounded stray dogs and cats with a license or microchip must be held at a shelter for ten days following impoundment, whereas animals without evidence of an owner must only be held for four days [36]. Animals in both categories may be killed or euthanized if “irremediably suffering”, a condition that the law does not define [36]. Licensing requirements for cats in the US continue to be uncommon.

Beyond the policies and procedures that govern shelter operations today, the pound model shapes how governmental animal shelters act and think as institutions, including the limitations of what shelters consider possible. This is not to say that every animal shelter in the contemporary United States is the same, but rather to draw attention to the starting point of the American pound as continuing to inform how governments and the public conceive of human–animal relationships and collective responsibilities to animals in US communities. In our work with shelters across the US, we have observed that path dependence makes it extraordinarily difficult for changes to take place and to remain in place: the accomplishments of a progressive shelter leader or local government administration are too often undone when their successors subscribe to the more regressive or punitive entrenched pound model, which is often the model in which they have been trained. Increases in the live release rate and other accomplishments won by a progressive leader or local government administration are often rapidly reversed or undone without justification when a new leader, whose philosophy and approach is grounded in the pound model, is the successor.

From the inception of pounds in the US, local governments emphasized capturing and killing as the primary way to address free-roaming dogs and cats. Over the course of the twentieth century, pounds became increasingly rationalized, making it easier for shelters to manage the population of animals in their care through systems of classification and surveillance. Housing animals in single-occupancy kennels, giving them identification numbers, and using logs and computer systems to track animals’ health and behavior are examples of rationalization, which values efficiency, calculability, predictability, and depersonalization.

Drawing on the pound model’s original commitment to the idea that free-roaming animals (especially dogs) are bodies out of place in a civilized society, local governments routinely stress that the role of animal services is to protect public safety, which means capturing stray and lost animals, and killing many of them. Local shelter practices are tied to local ordinances and these ordinances are informed by state statutes. In many cases, state statutes directly reflect beliefs about animals that are no longer widely accepted. For example, statutes in the state of Ohio specify that healthy animals can be killed after their stray hold, but only after the animal has been offered for 24 hours to a “nonprofit teaching or research institution or organization” [37]. The combination of state statutes, local ordinances, and local government administrations affirming death as the primary outlet for animals can make it difficult for animal shelters leaders to push back, especially when shelters are full.

Many government-run shelters continue to operate using hierarchical models of leadership that track closely with those of police departments, with staff holding job titles like Captain or Sergeant (animal service agencies funded by governments but operated by non-profit organizations appear less likely to adopt similar models). They also maintain cultures in which authority is not to be questioned, and where the public has little influence on shelter policies and practices. Most of the largest public animal shelter systems in the US do not have appointed advisory committees or other formalized oversight mechanisms. Because animal sheltering is considered a niche function and animal services employees are seen as having specialized skill sets and knowledge that exists apart from other government services, animal shelters are often left alone until someone files a public safety incident or welfare complaint against the shelter. If a shelter defers intake and a person or animal is injured, or if the population of animals in the shelter rises and there is a disease outbreak, local leadership is likely to intensify the pressure on the animal shelter director to kill for space or in the name of public safety [38].

Just as early pounds justified killing as necessary and often in the animals’ own best interests, contemporary discourses within many shelters support killing as a practice. One of the least contested and most widely accepted and repeated narratives within the animal sheltering industry is that shelters should guarantee the Five Freedoms, a set of principles that can be, and often are, used to justify killing. After public outcry in response to British animal advocate Ruth Harrison’s 1965 book on the conditions of farmed animals who were destined for slaughter [39], the British government assembled a committee to consider the welfare of animals in industrial agriculture. The ensuing report [40] included what came to be known as Five Freedoms, or that animals being raised for slaughter should be guaranteed the (1) freedom from hunger and thirst; (2) freedom from discomfort; (3) freedom from pain, injury, and disease; (4) freedom to express normal behavior; and (5) freedom from fear and distress [41].

Although the Five Freedoms were never meant to address the wellbeing of shelter animals, since the late twentieth century, the American Society for the Prevention of Cruelty to Animals (ASPCA), the Association of Shelter Veterinarians (ASV), American Humane, and other major companion animal welfare organizations in the US have adopted the Five Freedoms as the universal standard of care for animal shelters. In our extensive work in shelters, we find that the Five Freedoms are primarily conceptual and do not meaningfully exist in most animal shelters. Where practicing them is attempted, they exist only vaguely and in particular moments when staffing levels are high, motivated leadership is in place, and the intake of animals makes it possible to ensure basic welfare standards are met.

Freedom from death is not among the freedoms (although we would argue that freedom from baseless killing is necessary in order to experience any of the freedoms, and thus is the most basic freedom), and several of the freedoms can actually be—and, we have found, commonly are—used to justify shelter killing. For instance, if a shelter asserts that it has insufficient staffing to ensure each animal can express behavior normal for their species, the shelter can justify killing animals. Likewise, if an animal experiences discomfort due to a health issue like a respiratory infection, the shelter can justify killing. We have observed exactly these patterns time and again in animal shelters: the Five Freedoms become a way to assert that animals are better off dead.

More recently, animal shelters have started to adopt the terminology of the Five Domains, which was first described in 1994 as a reframing of the Five Freedoms to overcome some of their limitations and focus on positive life experiences instead of defining quality of life in negative terms (i.e., moving to language emphasizing access *to* rather than freedom *from*). The Five Domains, just like the Five Freedoms, were created to improve the welfare of animals in institutional settings, killed by slaughter or “euthanasia” before the end of their natural lives [42]. The core problem with the Five Freedoms and the Five Domains is that neither affirms the right to live. Instead, and in line with the pound model, the Five Freedoms and Five Domains accept killing as inherent to sheltering.

Along with the Five Freedoms, the newer Capacity for Care (C4C) philosophy ultimately justifies killing, even though it is specifically written for animal shelters. Capacity for Care is both an assertion that shelters must not exceed their capacity for care, which is determined by a complex and subjective series of calculations, and provides a justification for killing animals in shelters in excess of this number [43]. In a 2015 publication about C4C, the authors cite the ASV guideline statement that “every organization has a maximum capacity for care and the population in their care must not exceed that” [44]. Though this publication does recommend that shelters take steps to achieve live outcomes for animals that are not ill or dangerous, this message is secondary to the key point that there is a capacity that, “like Goldilocks and the Three Bears…is ‘just right’, not too big and not too small” [45]. While perhaps not originally intended to justify killing, the C4C approach lends itself very easily to be used in this way, and routinely is in public shelters.

While the Five Freedoms and the Five Domains uphold the longstanding culture of shelter killing by not addressing killing at all, the C4C model goes a step further by reifying the “anti-warehousing” logic that proponents of shelter killing have employed for decades. The anti-warehousing logic centers on the idea that animals who live in shelters for “too long” (a timeframe that is generally unspecified) suffer behavioral and psychological stress so severe that they are better off being killed than continuing to live in a shelter environment. Proponents of this perspective often assert that shelters are not intended to store animals, and use claims that long-term shelter stays are always inherently associated with animal suffering as a justification for killing. By extension, this implies that shelters are not quality environments for companion animals (contra, for instance, zoos and laboratories, where animals live in containment for their entire lives). Of course, shelters should provide impounded animals with enrichment, veterinary care, and opportunities for building relationships with other animals and humans, which most shelters are fully capable of doing but, as we have observed many times in large public shelters, choose not to. When shelter leaders or staff fall back onto the anti-warehousing logic, they typically do so to make excuses for killing. Critically engaging with the dominant narratives that uphold shelter killing illuminates that the entrenched belief system rooted in the pound model is always reactive, trapped inside its own self-limiting possibilities of how to solve the so-called problem of companion animals in our communities.

The claim that there are too many companion animals in the US to be moved through the animal shelter system and come out alive traces back to the first pounds and shelters, which viewed the companion animal population as unwieldy, hyper-reproductive, threatening to the development of a “civilized” and healthy nation, and problematic. Contemporary ideas about overpopulation maintain that there are too many unhoused companion animals to find homes for them all, and that the only way to reduce shelter populations is through the proactive sterilization of all cats and dogs and the killing of animals deemed surplus until the balance between supply and demand is met.

Even in this immensely challenging moment in animal sheltering, there are no data to suggest the US has an overall companion animal overpopulation problem (excluding possibly the population of community cats [46]). Rather, the issue is one of regional over- and under-supply of available animals, with supply exceeding demand in certain areas while demand exceeds supply in others [47,48,49]. In fact, there are multiple, complex factors that feed into killing impounded animals for space. These may include restrictive adoption practices, shelters being closed or mostly closed to the public, animals not being made available to the public to adopt in the first place, and the failure of shelters to compete within the emerging landscape of companion animal acquisition (e.g., failing to be competitive with breeders who sell animals online with no red tape and failing to utilize search engine optimization strategies that would bump sheltered animals up in search engine results). 

Overall, the number of dogs made available for adoption from shelters and rescues is not enough to meet the national demand for new dog acquisition [48]. When the animal sheltering industry perpetuates the idea that there are just too many animals [50], it affirms a narrative that data do not substantiate, and masks the problems that actually result in animals dying in shelters. Only by exploring why animals are dying in shelters can we identify and advocate for solutions. For example, Hassen has worked with the shelter system in San Antonio, Texas, where the animals who are killed are mostly healthy, friendly puppies and dogs [51]. San Antonio has a high rate of shelter impoundment, and seemingly low demand for shelter animals. Yet these same dogs would be considered highly adoptable in Boston, Massachusetts, a city with fewer available puppies and dogs relative to the human population. The problem is not that there are no homes for these animals but that there are barriers to getting them from San Antonio to Boston. 

Both anti-warehousing and overpopulation perspectives uphold the practice of shelter killing. The anti-warehousing position justifies shelter killing by maintaining that companion animals are better off dead than living in shelters. The overpopulation perspective excuses killing by implying that there simply are not enough homes for the animals needing homes and that these animals must be dealt with somehow (with the exception of some shelters with TNR programs, releasing impounded animals back into the community is never discussed).

These ideas also support each other: shelter managers and others in companion animal welfare routinely assert that animals are better off killed than being warehoused. The guidelines put forth by one major industry organization state that, “aversion to euthanasia is no excuse for poor welfare” [43]. In this language, there is an assumption that not killing contributes to problematic warehousing and that killing is both acceptable and necessary to maintain the health and welfare of the shelter population as a whole. These guidelines thus reflect the pound model and continue to reproduce it within contemporary shelters.

Efforts within the sheltering industry to improve the welfare of institutionalized animals are essential because poor welfare standards lead to conditions like disease and fighting that put animals’ lives at risk. They are also generally correlated with a poor quality of community service. However, positioning the killing of impounded animals as an acceptable outcome perpetuates institutional and public beliefs that uphold the problematic legacies of the pound model. In contrast, rejecting the killing of most animals in shelters outright and completely as an option opens up new possibilities for imagining and enacting versions of care that focus on companion animal guardian support, lost animal reunification, foster care programs, community involvement, innovations in adoption programs, and expediting permanent live outcomes as the central mechanisms through which shelters can avoid institutional overcrowding.

## 5. Invisibility and Transparency

Another legacy of the pound model is the lack of transparency within many shelters. Hundreds of new shelters were built during the post-war construction boom in the 1950s and 1960s, and though styles varied, most featured cages for individual animal housing, some of which are public-facing kennels and others of which are in non-public areas. These shelters were built to make the disposing of dead bodies as invisible and efficient as possible. This was, in fact, a desired outcome for late-nineteenth-century humane reformers: They wanted municipal animal control agents to stop killing animals in settings where the animals’ deaths could be seen, whether culling in the street or drowning in urban rivers, and to instead bring them to facilities to kill them outside of the public eye [15,16]. Many animal protectionists believed that witnessing the killing of animals would contribute to moral corruption [15]. They also believed animals should be killed as quickly and painlessly as possible. The system of killing and disposal was thus removed from the public gaze as shelters were routinely constructed in out-of-the-way areas, and animals were killed in a dedicated space that the public could not see or access.

Most animal shelters are still designed to move animals efficiently into and out of the system while controlling which animals the public can see and when. For instance, in the more than 125 government or government-contracted shelters the authors have visited, the area in which animal control officers park their vehicles to move animals from trucks to kennels is gated off so the public cannot see animals entering; staff routinely refer to this area as the sally port, a term that refers to the area where police or correctional officers unload new arrestees or convicts. Such a design is typically recommended; some shelter design guidelines even suggest making the process of owners surrendering animals invisible to prospective adopters [52,53]. Animals are promptly classified by intake type, setting them on specific paths through the shelter [5]. Stray and lost animals, who make up the majority of animals in shelters, are usually held in non-public viewing areas until they are available for adoption. Animals who come in with illnesses and injuries, if not euthanized immediately, are generally placed in medical kennels which the public cannot see or access. These non-public kennels are located proximal to the pre-euthanasia holding area and the euthanasia room, as are the mechanisms for disposing of the bodies, which might include incinerators, oil drums, refrigerated rooms, and/or conveyor belts. Although some shelters have worked to make all impounded animals visible, the design of shelters, animal shelter data-tracking systems, and the animal pathways set by shelter management reduce or eliminate transparency in many public shelters in the US today.

A lack of transparency extends beyond where the animals are housed and how they are moved through the shelter system. Many shelters do not provide public information about the circumstances of how the animals came into custody or how they have fared there. This is especially the case for animals who are not made available for adoption before being killed. Some shelters do not post all of the animals in their care on-line. Since COVID-19, some shelters have limited their visiting hours on a seemingly permanent basis. Public access to information about shelters can be difficult to access and/or decipher. Some provide statistical reports on-line only and solely in English. In order to understand shelter policies and practices around killing, members of the public must first figure out how many animals are dying, then attempt to discern the criteria for killing, then look at individual animal notes to find out what animals died, their stories, and why they were killed. Most of this information is only made available in response to a public records request under the Freedom of Information Act, and some shelters impose barriers that make it nearly impossible for advocates to obtain meaningful information. Due to a lack of standardized reporting and the tremendous variation in terminology, methodology, and definitions, it can be difficult to understand animal-level information, even if it is accessible. Keeping much of the work of animal shelters hidden from public view remains a key legacy of the pound model.

## 6. The Economics of Animal Sheltering

Like other aspects of the modern welfare state, the pound model emerged in part to help reduce the strains of the growth of capitalism and its attendant racial and class stratification, urbanization, and immigration [16]. Animal shelters help our society maintain a sense of social order, a social order in which free-roaming dogs especially are constructed as a threat to themselves and to public safety (free-roaming cats are often tolerated in communities despite anti-cat activism that blame cats for disease and negative environmental impacts [54]). Further, like shelters for unhoused people, shelters for animals manage a population deemed at least temporarily surplus in a capitalist economy. From a critical political economic perspective [55,56], animal shelters can be understood as providing a taxpayer-funded catch pan for corporations, landlords, banks, real estate developers, and investors, who are responsible for much of the displacement and precarity that results in animals coming into shelters. Shelters provide a hidden-from-public-view, socially acceptable repository for housing animals who are displaced because the conditions of the current economic systems cannot accommodate them. The existence of animal shelters perpetuates and upholds housing discrimination against animals and the people who care for them, because shelters make it possible to establish bans or restrictions on certain breeds, species, or sizes of animals.

Like prisons [57], shelters are another way for the state to try to fix a crisis of capitalism, namely the ongoing displacement of people and companion animals and the continued maintenance of a surplus underclass essential to keeping wages low. People who are not housing-insecure or incarcerated are disciplined into ways of living and thinking that certain forms of protection and security are legitimate. Unhoused animals, like other so-called surplus populations, including unhoused people and carcerally impacted people, are spun into gold through what abolitionist and disability justice scholar Liat Ben-Moshe [58] refers to as “clever capitalist alchemy”. This happens when capitalism extracts value from the abandonment of entire populations, such as disabled people, unemployed people, poor people of color, and unhoused animals. Unhoused animals who have been displaced through the routine processes of capitalism have given rise to an entire industry of shelters, humane societies, rescues, and philanthropies of all sizes.

Animal shelters operate within the broader economic system, reflecting how the system itself values and de-values animals and human–animal relationships [59,60,61]. Some animals—such as those who are younger, appear to be “purebred”, and who have no known health or behavior issues—have economic value, and are more likely to exit shelters alive. The rest of the animals in shelters are without value or may even have negative value. To be clear, this system of valuing animals operates both in and out of the shelter: in a society in which companion animals are commodified, or viewed as products that can be bought and sold [60,61,62], it is not the shelter alone establishing the differential value of various groups of animals. Ultimately, the commodification of animals within our capitalist system has resulted in competition among rescue groups, adopters, and private shelters for the small percentage of animals with the highest perceived value. Open-admission government shelters responsible for taking in all stray and surrendered animals are tasked with the assessment, sorting, and distribution of animals, extracting profit where possible and killing less desirable animals-as-products in order to make room for the endless stream of both commodifiable and “worthless” animals that are yet to enter.

Just as importantly, the separation of animals from their communities and caregivers is entrenched in the punitive logics that guide many services, and residents are expected to pay for public services and face financial penalties for many minor transgressions [63,64]. This combination of requiring people to pay fees and fines or facing penalties (and the loss of their animal to the shelter system) has also been part of the pound model since its inception in the US. The system of fines and fees has evolved to include additional fees like kennel permits and fines for failing to abide by any mandatory measures like spay/neuter requirements. Animals who are unsterilized or unlicensed are often subject to an entirely different and higher fee structure than sterilized or licensed animals, which can make it very difficult for their caregiver to reclaim them. Fees vary from community to community and state to state, and are largely within the purview of local agencies [65]. In the contemporary US, impoundment and kenneling fines can be hundreds or even thousands of dollars, depending on the circumstances and duration of the impound and who is connected to a particular animal. Many states support civil forfeiture statutes in cases of neglect, abuse, or hoarding, or even when a guardian is charged with an offense not directly related to the animal. This allows law enforcement officials to force owners to “pay to play”, paying impoundment and care fees so the guardian maintains legal custody through the duration of a court case. These fees are often thousands of dollars, and for owners who cannot pay, their animal can simply be permanently removed from their custody and be killed, transferred, or adopted out by the shelter without the owner having any right to know the outcome.

Also, like some other aspects of the modern welfare state, pounds started out as local institutions, and overwhelmingly remain so. Contemporary animal service agencies and organizations are typically funded through a combination of internally generated fees and allocations from local taxes, and sometimes also from philanthropic support. This model of mixing public and private funds, taxpayer-generated, and fine- and fee-generated funds, has been in place since the late nineteenth century. Today, this localization of the pound model and its funding continues to contribute to substantial resource disparities across shelters.

Animal services budgets represent only a tiny fraction of the overall city, county, or town budget, despite the fact that around 60% of U.S. residents claim guardianship of one or more companion animals [66]. Animal services are generally underfunded relative to community centers, parks, public safety, and health departments. Many municipalities rely on nonprofit groups to take animals with no financial support, in some cases even charging them fees to rescue shelter animals.

What we consistently observe across animal shelters is that the resources and standards available to animals and to humans in a shelter strongly correlate with the socioeconomic status and racial/ethnic background of the residents the shelter serves. In affluent communities, where the population tends to be predominantly white, animal shelters are often non-profit organizations that hold government contracts, and typically benefit from large philanthropic gifts and high levels of community donor support, as well as from generous government contract terms for services like licensing, ordinance enforcement, and the rehoming of shelter animals [5]. In poor communities, where more of the population is likely to be made up of people who are Black, Latinx, Native American, or belong to other minority groups, shelters are more likely to be public facilities with limited, if any, philanthropic support, and cost-focused contracts that compensate the shelter the bare minimum for services provided. Disparities also exist among shelters in various cities and regions of the United States regarding the size of the population of animals entering shelters. In southern cities, animal intakes are often above 15,000 annually, whereas intakes in northern areas, particularly the northeast and northwest, tend to be significantly smaller.

Given these general trends, animals who are impounded in more affluent communities may have better experiences of impoundment, with larger kennels, more enrichment programs, proactive reunification and adoption programs, and low rates of disease transmission [5]. Animals who are impounded in shelters serving lower-income communities are often housed in shelters built 50 years ago or more, with little or no enrichment, limited programs focused on reunification or adoption, lack of access to protective vaccines, and higher rates of disease transmission, which is problematic because shelter staff can use even symptoms of non-life-threatening diseases like upper respiratory infections as a basis for justifying killing an animal. Shelters in affluent communities are more likely to have funding to support outreach efforts, active volunteer programs, and an array of services, including animal behavior and training, that are available to the human public. Shelters in poorer communities may be limited in their capacity to offer foster, volunteer, and adoption programs, and provide critical community outreach services. Shelters in poorer communities are routinely crowded and are most likely to kill animals as part of their regular, daily operations in order to maintain a predetermined capacity.

Certainly, there are some affluent government-contracted nonprofit animal shelters that have relatively higher rates of killing than their poorer counterparts due to competing philosophical orientations towards lifesaving and how agencies set priorities and measure success. However, the pattern remains that animals in shelters with more resources have better experiences and outcomes. In a recent analysis of data collected by Shelters Animal Count, one of the most comprehensive data sources for information about the intakes and outcomes of animals in US animal shelters, Laura A. Reese found that, “Areas with greater economic stress and lower educational attainment are more likely to have a municipal shelter, which increases stray intake, and ultimately euthanasia. Community economic stress is also directly associated with lower adoption and higher euthanasia rates” [67].

## 7. Reconciling the Pound Past

The legacy of the pound model continues in animal sheltering in the US in four primary ways. First, even with major reductions in killing, the institutional culture of most public animal shelters endorses killing as a necessary aspect of companion animal welfare. Second, as pounds worked to hide killing from the public, so, too, do contemporary public shelters. Third, animal sheltering and control agencies and organizations continue to rely on confinement and caging, and all too often use the conditions of confinement as a justification for killing. Finally, the localized pound model reflects wealth disparities across communities because of its primary support from local taxes and fees; this model continues today.

For many decades, animal sheltering in the US has been making incremental changes. These changes typically emphasize proactive spay and neutering and other prevention efforts, improving the quality of the experience of impounded animals, and increasing the percentage of animals who leave alive. Over the past fifty years, the number of animals impounded and number killed in US shelters has decreased substantially, thanks both to changes inside of shelters and changes outside of them [49,68]. However, many long-standing underlying problems continue to plague shelters, even among those that express a commitment to achieving live outcomes for the majority of animals that enter their custody.

Despite widespread reform efforts over the past fifty years, the animal sheltering model in the US has never departed from the foundations of the pound model in the late 1800s and early 1900s. The path set in motion more than 150 years ago continues to handily shake off efforts to disrupt it. The current system is unable to move beyond its roots of separation, confiscation, caging, and killing. Underneath the interventions that have positively impacted intake and live release numbers, the basis for operation remains a historically and geographically specific intolerance of free-roaming animals, a refusal to address the interests of animals, the normalization of state-sanctioned severing of the human–animal bond, and the systematic killing of cats and dogs. When the system fails to operate at maximum efficiency, and when shelters find themselves with a temporary surplus of animals, killing presents itself as the only feasible response within our current framework, as happened in the US in 2023.

Of course, there have been moments when US shelters could have moved in a different direction and stepped off the pound pathway like some other parts of the world did, following a community-centered of animal welfare that centers interspecies connection and health. Instead of investing in the systems for the mass killing of animals believed to be stray, or continuing to build new shelters, shelter entrepreneurs could have taken action to affirm the rights of animals to live in our cities and towns. Instead of disproportionately policing poor communities and communities of color and using norms around animal keeping as indicators of humans’ social worth [5,29], shelters could have established interventions to support both people and their animal companions, wherever they lived.

Envisioning a different future begins with reckoning with our past and present. The dominant approach to companion animal welfare in the US does not have to continue—the sheltering industry and our societal tolerance for animals living among us *can* entirely change direction. The companion animal welfare industry is plastic: even if it has been following a particular pathway, the path is not permanently fixed. US shelters could instead move towards a new vision of animal welfare in which impoundment is no longer the first response to free-roaming companion animals and in which death is no longer an acceptable response to animals finding themselves in what is currently constructed as the wrong place at the wrong time. If we assert *seven* freedoms, including the right to not be killed due to a lack of space or resources, alongside the right not to be discriminated against based on species, breed, size, color, or other characteristics, we would innovate new ways of doing things that could lead us towards a new system [5]. We can begin to envision a different relationship between people and animals, a relationship in which animals are not understood, legally or socially, as property [69,70] and in which supporting human and animal well-being and togetherness is the central goal of both human-centered (e.g., social services, family services) and animal-centered (e.g., animal services) programs and offices. While detailing the precise steps to such a future is beyond the scope of this paper, we note here that a model of togetherness for humans and companion animals would entail abolishing animal shelters as we know them today. It would further involve embracing principles from multispecies democracy so that animals’ interests and perspectives are incorporated into their care [70,71], incorporating community members into envisioning and enacting programs of care, and redefining the human–animal relationship as a non-property relationship.

To get there, we need to look to alternative models of relationships between humans and companion animals, being open to learning lessons from diverse approaches both in and outside of the US that can be used to inform a new path forward. Other pathways, which are not yet imagined or in practice, can also be envisioned if we commit to turning away from accepting how things are and instead think instead about beginning anew. A critical first move is to stop accepting that animals must die in shelters due to a lack of space or resources. If this commitment serves as a starting point, innovation and evolution will follow. It is possible to achieve a society in which taking animals out of their homes and communities, caging, and killing them is no longer an acceptable series of practices. Identifying and rejecting the historical legacies of the pound model is the first step in leaving the path it has set for us.

## Data Availability

No new data were created or analyzed in this study. Data sharing is not applicable to this article.

## References

[1] Hubka T.C. (2004). Big House, Little House, Back House, Barn: The Connected Farm Buildings of New England.

[2] Pierson P. (2000). Increasing Returns, Path Dependence, and the Study of Politics. Am. Political Sci. Rev..

[3] Shelter Animals Count Newly Released Animal Sheltering Data Shows Shelters Urgently Need Community Support; Dog Euthanasia Increasing as More Dogs Enter Shelters than Leave. https://www.shelteranimalscount.org/newly-released-animal-sheltering-data-shows-shelters-urgently-need-community-support-dog-euthanasia-increasing-as-more-dogs-enter-shelters-than-leave/.

[4] Shelter Animals Count Nearly 245,000 Additional Pets in the Shelter System This Holiday Season. https://www.shelteranimalscount.org/estimated-245000-additional-dogs-and-cats-in-the-shelter-system-this-holiday-season/.

[5] Guenther K.M. (2020). The Lives and Deaths of Shelter Animals.

[6] Guenther K.M., Guenther K.M., Keenan J.P. (2023). Working Toward Livable Lives through Reproductive Justice for Community Cats. When Animals Die: Examining Justifications and Envisioning Justice.

[7] Guenther K.M. (2017). Volunteers’ Power and Resistance in the Struggle for Shelter Animal Survival. Sociol. Forum.

[8] Guenther K.M. (2023). Challenging and Reinforcing the Ability/Disability System through Advocacy for Disabled Dogs. Disabil. Stud. Q..

[9] Best Friends Animal Society Lifesaving by the Numbers: Animal Welfare Statistics. https://resources.bestfriends.org/article/lifesaving-numbers-animal-welfare-statistics.

[10] Rowan A.N. (2018). Companion Animal Statistics in the USA. Demogr. Stat. Companion Anim. Popul..

[11] Count S.A. 6 Data Don’ts in 2023. https://www.shelteranimalscount.org/6-data-donts-in-2023/.

[12] Merriam-Webster (2024). Merriam-Webster Dictionary. https://www.merriam-webster.com/dictionary/euthanasia.

[13] American Humane Association (2004). Asilomar Accords: Saving Lives Not Pointing Fingers. https://www.americanhumane.org/publication/asilomar-accords-saving-lives-not-pointing-fingers/.

[14] Armstrong M.C., Tomasello S., Hunter C., Salem D.J., Rowan A. (2001). A Brief History of Shelters and Pounds. The State of the Animals.

[15] Unti B.O. (2002). The Quality of Mercy: Organized Animal Protection in the United States, 1866–1930.

[16] Pearson C. (2021). Dogopolis: How Dogs and Humans Made Modern New York, London, and Paris.

[17] Wetzel H.M. (2019). Mangy Curs and Stoned Horses: Animal Control in the Districtof Columbia from the Beginnings to about 1940.

[18] Freeberg E. (2020). A Traitor to His Species: Henry Bergh and the Birth of the Animal Rights Movement.

[19] (1877). New York Times. Drowning the Dogs. Over Seven Hundred Unlicensed Disposed of Scenes at the Pound Statistics for the Week. New York Times.

[20] Lee J. (2008). Where They Used to Drown the Dogs. New York Times.

[21] Houser S.K. (2018). Prodigal Pets: A History of Animal Sheltering in America and the Origin of the No Kill Movement.

[22] Jones S.D. (2002). Valuing Animals: Veterinarians and Their Patients in Modern America.

[23] Davis J.M. (2016). The Gospel of Kindness: Animal Welfare and the Making of Modern America.

[24] Women’s Animal Center (2023). History of Women’s Animal Center. Philadelphia, PA. https://womensanimalcenter.org/about-us.

[25] Finnegan C.J., Brookes S.M., Johnson N., Smith J., Mansfield K.L., Keene V.L., McElhinney L.M., Fooks A.R. (2022). Rabies in North America and Europe. J. R. Soc. Med..

[26] Centers for Disease Control and Prevention Human Rabies. https://www.cdc.gov/rabies/location/usa/surveillance/human_rabies.html.

[27] Krebs J.W., Noll H.R., Rupprecht C.E., Childs J.E. (2002). Rabies Surveillance in the United States during 2001. J. Am. Vet. Assoc..

[28] Nadal D. (2020). Rabies in the Streets: Interspecies Camaraderie in Urban India.

[29] Arluke A., Rowan A. (2020). Underdogs: Pets, People, and Poverty.

[30] County of Riverside (2023). Riverside County Code. https://library.municode.com/ca/riverside_county/codes/code_of_ordinances?nodeId=TIT6AN_CH6.04ANGE_6.04.040IMAN.

[31] Smith L.M., Hartmann S., Munteanu A.M., Dalla Villa P., Quinnell R.J., Collins L.M. (2020). The Effectiveness of Dog Population Management: A Systematic Review. Animals.

[32] Shelter Animals Count Intake and Outcome Database. https://www.shelteranimalscount.org/intake-and-outcome-database-iod/.

[33] Wisch R.F., Dillingham A. (2017). Table of State Holding Laws.

[34] Best Friends Animal Society (2020). The State of US Animal Sheltering, 2020. https://network.bestfriends.org/research-data/research/state-us-animal-sheltering-2020.

[35] (2019). Best Friends Animal Society The State of US Animal Sheltering, 2019. https://network.bestfriends.org/research-data/research/state-us-animal-sheltering-2019.

[36] Hayden T.E., O’Connell J. (1998). SB 1785: The Hayden Act. http://www.leginfo.ca.gov/pub/97-98/bill/sen/sb_1751-1800/sb_1785_bill_19980923_chaptered.html.

[37] Legislative Service Commission (State of Ohio) (2012). Disposing of Impounded Dogs. https://codes.ohio.gov/ohio-revised-code/section-955.16/9-10-2012.

[38] Ramirez M. ‘Nationwide Crisis’: Facing Lower Adoption Rates, Some Shelters Reconsidering Euthanasia Policies. USA Today 2022. https://www.usatoday.com/story/news/nation/2022/12/22/animal-shelters-overcrowding-prompts-some-revisit-euthanasia/10933722002/.

[39] Harrison R. (2013). Animal Machines: The New Factory Farming Industry.

[40] The Technical Committee to Inquire into the Welfare of Animals Kept under Intensive Livestock Husbandry Systems (1965). Report of the Technical Committee to Inquire into the Welfare of Animals Kept under Intensive Livestock Husbandry Systems.

[41] Association of Shelter Veterinarians The Five Freedoms. https://www.sheltervet.org/five-freedoms.

[42] Mellor D.J., Beausoleil N.J., Littlewood K.E., McLean A.N., McGreevy P.D., Jones B., Wilkins C. (2020). The 2020 Five Domains Model: Including Human-Animal Interactions in Assessments of Animal Welfare. Animals.

[43] Association of Shelter Veterinarians Guidelines for Standards of Care in Animal Shelters. https://www.sheltervet.org/guidelines-for-standards-of-care-in-animal-shelters.

[44] UC Davis Koret Shelter Medicine Program Overview of Capacity for Care (C4C). https://www.sheltermedicine.com/library/resources/?r=overview-of-capacity-for-care-c4c.

[45] UC Davis Koret Shelter Medicine Program Calculating Shelter Capacity. https://www.sheltermedicine.com/library/resources/?r=calculating-shelter-capacity.

[46] Kass P.H., Rochlitz I. (2007). Cat Overpopulation in the United States. The Welfare of Cats.

[47] Dangler M. (2023). 2022 Pima Animal Care Center—Safety Report. https://content.civicplus.com/api/assets/a57eea3d-3405-421b-85e6-94827bb3dc7a?cache=1800.

[48] Cushing M. (2021). Pet Nation: The Inside Story of How Companion Animals Are Transforming Our Homes, Culture, and Economy.

[49] Marsh P. (2010). Replacing Myth with Math: Using Evidence-Based Programs to Eradicate Shelter Overpopulation.

[50] People for the Ethical Treatment of Animals Companion Animal Overpopulation. https://www.peta.org/issues/animal-companion-issues/overpopulation/.

[51] City of San Antonio Animal Care Services Urgent Pet Placement Reports. https://www.sa.gov/Directory/Departments/ACS/Placement-Surrender/Urgent.

[52] Schlaffer L., Bonacci P., Miller L., Zawistowski S. (2013). Shelter Design. Shelter Medicine for Veterinarians and Staff.

[53] (2016). American Humane Operational Guide: Planning and Building an Animal Shelter. https://www.americanhumane.org/publication/animal-shelter-operational-guide-planning-and-building-an-animal-shelter/.

[54] Marra P.P., Santella C. (2016). Cat Wars: The Devastating Consequences of a Cuddly Killer.

[55] Nibert D.A. (2002). Animal Rights/Human Rights: Entanglements of Oppression and Liberation.

[56] Torres B. (2007). Making A Killing: The Political Economy of Animal Rights.

[57] Gilmore R.W. (2022). Abolition Geography: Essays towards Liberation Abolition Geography: Essays Towards Liberation.

[58] Ben-Moshe L. (2020). Decarcerating Disability Deinstitutionalization and Prison Abolition.

[59] Schukin N. (2009). Animal Capital: Rendering Life in Biopolitical Times.

[60] Collard R.-C. (2020). Animal Traffic: Lively Capital in the Exotic Pet Trade.

[61] Collard R.-C., Dempsey J. (2013). Life for Sale? The Politics of Lively Commodities. Environ. Plan. A.

[62] Haraway D. (2008). When Species Meet.

[63] Desmond M. (2023). Poverty, By America.

[64] Swain A.E., Noblit G.W. (2011). Education in a Punitive Society: An Introduction. Urban Rev..

[65] People for the Ethical Treatment of Animals List of Current Spay-and-Neuter Ordinances. https://www.peta.org/issues/animal-companion-issues/overpopulation/spay-neuter/current-spay-and-neuter-ordinances-list/.

[66] American Veterinary Medical Association (2022). 2022 AVMA Pet Ownership and Demographics Sourcebook.

[67] Reese L.A. (2022). Community Factors and Animal Shelter Outcomes. J. Appl. Anim. Welf. Sci..

[68] Rowan A., Kartal T. (2018). Dog Population & Dog Sheltering Trends in the United States of America. Animals.

[69] Deckha M. (2021). Animals as Legal Beings: Contesting Anthropocentric Legal Orders.

[70] Donaldson S., Kymlicka W. (2013). Zoopolis: A Political Theory of Animal Rights.

[71] Meijer E. (2019). When Animals Speak: Towards an Interspecies Democracy.

